# Management of Dyslipidemia in Patients with Non-Alcoholic Fatty Liver Disease

**DOI:** 10.1007/s11883-022-01028-4

**Published:** 2022-05-04

**Authors:** Anna Martin, Sonja Lang, Tobias Goeser, Münevver Demir, Hans-Michael Steffen, Philipp Kasper

**Affiliations:** 1 Clinic for Gastroenterology and Hepatology, Faculty of Medicine - University Hospital Cologne, University of Cologne, Kerpener Str. 62, 50937 Cologne, Germany; 2 Department of Hepatology and Gastroenterology, Campus Virchow Clinic, Charité University Medicine, Berlin, Germany; 3 Hypertension Center, Faculty of Medicine - University Hospital Cologne, University of Cologne, Cologne, Germany

**Keywords:** Dyslipidemia, ASCVD, Cardiovascular risk, NAFLD, LDL-cholesterol, Hypertrigylceridemia

## Abstract

**Purpose of Review:**

Patients with non-alcoholic fatty liver disease (NAFLD), often considered as the hepatic manifestation of the metabolic syndrome, represent a population at high cardiovascular risk and frequently suffer from atherogenic dyslipidemia. This article reviews the pathogenic interrelationship between NAFLD and dyslipidemia, elucidates underlying pathophysiological mechanisms and focuses on management approaches for dyslipidemic patients with NAFLD.

**Recent Findings:**

Atherogenic dyslipidemia in patients with NAFLD results from hepatic and peripheral insulin resistance along with associated alterations of hepatic glucose and lipoprotein metabolism, gut dysbiosis, and genetic factors.

**Summary:**

Since atherogenic dyslipidemia and NAFLD share a bi-directional relationship and are both major driving forces of atherosclerotic cardiovascular disease (ASCVD) development, early detection and adequate treatment are warranted. Thus, integrative screening and management programs are urgently needed. A stepwise approach for dyslipidemic patients with NAFLD includes (i) characterization of dyslipidemia phenotype, (ii) individual risk stratification, (iii) definition of treatment targets, (iv) lifestyle modification, and (v) pharmacotherapy if indicated.

## Introduction

Non-alcoholic fatty liver disease (NAFLD) has become the most common chronic liver disease in Western countries and currently affects up to 25% of the general adult population. Due to its close association with type 2 diabetes mellitus (T2DM), hypertension, and dyslipidemia, it is often considered as the hepatic manifestation of the metabolic syndrome [1–3]. NAFLD encompasses a spectrum of liver disorders ranging from simple hepatocellular steatosis (non-alcoholic fatty liver, NAFL) to inflammatory non-alcoholic steatohepatitis (NASH) with or without concomitant fibrosis [4–6].

All-cause mortality is higher in patients with NAFLD compared to subjects without NAFLD, with the most common causes of death being cardiovascular disease (CVD), extrahepatic cancers, and liver-related complications [7–9].

In particular, atherosclerotic cardiovascular disease (ASCVD) represents a major disease burden in the NAFLD population and accumulating evidence indicates that NAFLD has to be considered as a significant risk factor for fatal or non-fatal CVD events [10–13, 14•].

Another important risk factor in this context is atherogenic dyslipidemia, characterized by plasma hypertriglyceridemia, increased triglyceride-rich lipoproteins including very-low-density lipoproteins (VLDL), and their remnants intermediate-density lipoproteins (IDL), a predominance of small dense low-density lipoprotein (sd-LDL) particles together with low high-density lipoprotein (HDL) cholesterol levels [15, 16].

Results from imaging studies demonstrate signs of subclinical arherosclerosis to be present in approximately 70% and 45% of middle-aged men and women, respectively, and low-density lipoprotein (LDL) has globally been recognized as the major driving force in the development of ASCVD and its clinical manifestations [17, 18].

Atherogenic dyslipidemia can be observed in a large majority of patients with NAFLD and results from hepatic and peripheral insulin resistance along with associated alterations of the hepatic glucose and lipoprotein metabolism, gut dysbiosis, and genetic factors [4, 16].

Since atherogenic dyslipidemia may be at least partially responsible for the increased subclinical and clinical ASCVD burden in patients with NAFLD, it represents a central treatment target in this population with high cardiometabolic risk [10, 11, 13, 19].

In 2020, a change of the terminology from NAFLD to metabolic associated fatty liver disease (MAFLD) has been proposed [20–22] and the diagnosis can be made in the presence of hepatic steatosis and at least one of the following criteria: (i) overweight or obesity, (ii) T2DM, and (iii) metabolic dysregulation (at least two factors among increased waist circumference, hypertension, hypertriglyceridemia, low serum HDL-cholesterol levels, impaired fasting plasma glucose, insulin resistance, or subclinical inflammation evaluated by high-sensitivity C-reactive protein (CRP) levels). While there is still an ongoing debate, the term NAFLD is maintained in this narrative review that describes the relationship between NAFLD and dyslipidemia, delineates interrelated pathophysiological mechanisms in the development of ASCVD, and focuses on up-to-date management and treatment approaches for dyslipidemic patients with NAFLD.

### Prevalence of Dyslipidemia Among Patients with NAFLD

Information about the frequency of lipid disorders among patients with NAFLD involving changes in serum cholesterol (hypercholesterolemia), triglycerides (hypertriglyceridemia), or both (combined dyslipidemia) varies. In a recent comprehensive meta-analysis of Younossi et al. including 86 studies with information about 8,515,431 patients with NAFLD from 22 countries, the overall prevalence of combined dyslipidemia in patients with NAFLD or NASH was estimated to be 69% and 72%, respectively [3]. This is in line with findings from the REGENERATE study that investigated the effects of obeticholic acid as potential treatment option in NAFLD, where 68–70% of a total of 931 recruited patients with NAFLD displayed combined dyslipidemia at baseline [23].

Hypercholesterolemia, as a specific subtype, was found in 44% of patients with NAFLD in a recent large prospective cohort study including middle-aged US adults [24]. In patients with NASH, prevalence rates of hypercholesterolemia are even higher and range from 60 up to 90% [25, 26].

Focusing on hypertriglyceridemia, the overall prevalence among patients with NAFLD or NASH has been reported in a recent meta-analysis to be 41 and 83%, respectively. These rates are confirmed by other studies and it has been shown that in particular patients with NASH as well as patients with NAFLD with concomitant T2DM are more frequently affected [27–30].

### Pathogenesis of Dyslipidemia in NAFLD

Alterations in lipid metabolism are centrally involved in both NAFLD and ASCVD development. In NAFLD pathogenesis, accumulation of liver fat results from a dysbalance between different pathways: inadequate uptake of circulating lipids, increased hepatic de novo lipogenesis (DNL), insufficient enhancement of fatty acid oxidation, and altered export of lipids as components of VLDL [4, 31]. An elevated hepatic uptake of lipids in combination with enhanced DNL leads to an increase triglyceride synthesis and elevated secretion of VLDL [16]. This overproduction of VLDL initiates an atherogenic dyslipidemic milieu including plasma hypertriglyceridemia, accumulation of triglyceride-rich lipoproteins, increased number of sd-LDL particles, and low HDL-cholesterol levels [4, 17, 32]. Apolipoprotein B containing triglyceride-rich lipoproteins and their remnants (IDL) as well as the genetically determined cholesterol-rich Lp(a), composed of apolipoprotein (B) and (a), are an essential part of the development of atherosclerosis. Following the infiltration of the subendothelial space of the vascular wall, lipoproteins act as damage-associated molecular patterns (DAMPs) and lead to an activation of Toll-like receptors (TLR), which play a crucial role in the innate immune response.

Especially triglyceride-rich lipoproteins containing apolipoprotein C3 (ApoC3) are important activators of TLRs 2 and 4, which leads to activation of the NLRP3 (NOD-like receptor family, pyrin domain-containing protein 3) inflammasome [32, 33]. Subsequently, NLRP3 inflammasome activation causes an activation of the enzyme caspase-1 (Interleukin-1 (IL) converting enzyme), followed by cleavage and thus activation of the IL-1ß family with subsequent induction of the IL-1 to IL-6 to CRP pathway. This inflammatory pathway plays a central role in ASCVD and vascular inflammation and is frequently activated in patients with NAFLD [4, 34]. Interestingly, certain metabolic environments such as an increased hepatic triglyceride pool or genetic alterations, which are both associated with NAFLD, modulate the formation of atherogenic ApoB lipoproteins, which promotes a more proinflammatory phenotype [32].

Insulin resistance is another important driver for the lipoprotein abnormalities in NAFLD. It affects several processes such as dyslipidemia, hyperglycemia, activation of oxidative stress and inflammation, endothelial dysfunction, and ectopic lipid accumulation, altogether stimulating ASCVD development [16, 35]. A recent study showed that alterations in lipid metabolism were mainly related to measures of insulin sensitivity rather than to obesity or the presence of NASH [36].

In conclusion, accumulating evidence indicates that NAFLD-driven dyslipidemia is substantially involved in the development of ASCVD in this at risk population [16].

### Management Approaches of Dyslipidemia in Patients with NAFLD

Since patients with both NAFLD and dyslipidemia are at substantial ASCVD risk, integrative screening programs are urgently needed and forced management of dyslipidemia plays a pivotal role in the primary and secondary prevention of ASCVD. The management of dyslipidemia in patients with NAFLD should be performed according to recent guideline recommendations, e.g., the European Society of Cardiology (ESC) / European Atherosclerosis Society (EAS) guidelines for the management of dyslipidemia or the recently published ESC guidelines on cardiovascular disease prevention in clinical practice [37–39].

General principles of managing dyslipidemia in patients with NAFLD include the following: (i) diagnosis and characterization of dyslipidemia, (ii) ASCVD risk stratification of patients based on recommended criteria and modern scoring algorithms (e.g., SCORE2, ACC/AHA ASCVD risk estimator), (iii) definition of treatment targets for serum lipids, (iv) lifestyle modification, and (v) pharmacotherapy if indicated.

### Treatment Approaches of Dyslipidemia in Patients with NAFLD

#### Diagnosis and Characterization of Dyslipidemia

An individualized diagnosis of dyslipidemia involves a comprehensive baseline assessment of the lipid profile including the determination of the following parameters: total cholesterol, LDL-cholesterol, non-HDL-cholesterol, HDL-cholesterol, and triglycerides (the latter in the fasting state when using the Friedewald formula to estimate LDL-cholesterol). ApoB analysis, if available, is recommended for risk assessment, particularly in people with high triglyceride levels, DM, obesity, metabolic syndrome, or very low LDL-cholesterol levels. Lp(a) measurement should be considered at least once in each adult persons’ lifetime to identify those with very high inherited Lp(a) levels > 180 mg/dL (> 430 nmol/L) who may have a lifetime risk of ASCVD equivalent to the risk associated with heterozygous familial hypercholesterolemia.

#### Individual Risk Stratification

After the assessment of an abnormal lipid profile, an individual risk stratification of the total ASCVD risk should be performed in each patient. This includes a systematic global assessment of CVD risk factors, including documented manifestations of ASCVD (e.g., coronary or carotid artery plaques), blood pressure, history of cigarette smoking, type 1 or type 2 DM, overweight or obesity as measured by the body mass index (BMI), and waist circumference, chronic kidney disease (CKD), family history of premature CVD (men < 55 years and women < 60 years), and genetic lipid disorders (e.g., familial hypercholesterolemia) [40].

Furthermore, it is useful to evaluate additional factors modifying individuals ASCVD risk such as physical inactivity, alcohol intake, psychosocial stress (incl. vital exhaustion or social deprivation), chronic immune-mediated inflammatory disorders, psychiatric disorders, cardiac arrhythmias (e.g., atrial fibrillation), and obstructive sleep apnea syndrome [40].

In addition, an estimation of the 10-year CVD risk can be performed [41]. For this purpose, current European guidelines recommend the recently published SCORE2 algorithm for people < 70 years of age and the SCORE2-OP (older people) algorithm for individuals ≥ 70 years of age [39, 42]. The SCORE2 algorithm estimates the individual 10-year risk of fatal and non-fatal CVD events in apparently healthy people aged 40–69 years with CVD risk factors that are untreated or have been stable for several years [39].

Although there are no specific ASCVD risk prediction tools that take into account the presence or severity of NAFLD, using a risk prediction algorithm can help to identify patients with NAFLD at higher risk of CVD who should benefit most from preventive action [43•, 44].

In a recent study by Golabi et al., it has been shown that an elevated ASCVD risk score ≥ 7.5% (defining an intermediate 10-year risk) among patients with NAFLD is associated with a higher risk of overall and cardiovascular mortality, confirming the usefulness of such scoring systems to identify patients at risk in this population [43•].

Validated cardiovascular risk calculators, such as *SCORE2*, typically divide the individual 10-year CVD risk in different categories (e.g., low, moderate, high, and very high risk).

While, for example, patients with well-controlled short-standing DM (e.g., < 10 years) without evidence for target organ damage or additional ASCVD risk factors were classified as patients with moderate risk, patients with additional risk factors such as CKD, genetic lipid disorders, elevated blood pressure, or already established ASCVD are considered as patients at high or very high CVD risk [39].

Due to the fact that NAFLD has been recognized as significant risk factor for CVD morbidity and mortality with an approximate 10-year CVD risk ranging from 5 to 22% in recent trials [45, 46], it seems reasonable to classify the risk for patients with NAFLD at least according to that of patients with DM [47]. This means, that patients with NAFLD without any other additional ASCVD risk factor (e.g., arterial hypertension, smoking, DM, obesity, CKD) should be classified as low to moderate CVD risk, whereas patients with NAFLD and at least one additional ASCVD risk factor should be classified as high CVD risk. Similarly, patients with NAFLD with markedly elevated single risk factors, in particular total cholesterol > 310 mg/dL (8 mmol/L) or LDL-cholesterol > 190 mg/dL (4.9 mmol/L) or familial hypercholesterolemia should be classified as high CVD risk.

Patients with NAFLD and documented clinical manifestations of ASCVD, including previous acute myocardial infarction, coronary revascularization or other arterial revascularization procedures, stroke or transient ischemic attack, aortic aneurysm or peripheral artery disease, and patients with NAFLD and documented ASCVD on imaging, including plaques on coronary angiography, carotid ultrasound, or on computed tomography angiography (CTA), should be classified as very high CVD risk individuals*.*

In addition, patients with NAFLD and advanced CKD (e.g., eGFR 45–59 mL/min/1.73 m^2^ and microalbuminuria or eGFR < 45 mL/min/1.73 m^2^ irrespective of albuminuria) should also be classified as very high CVD risk.

**Fig. 1:**
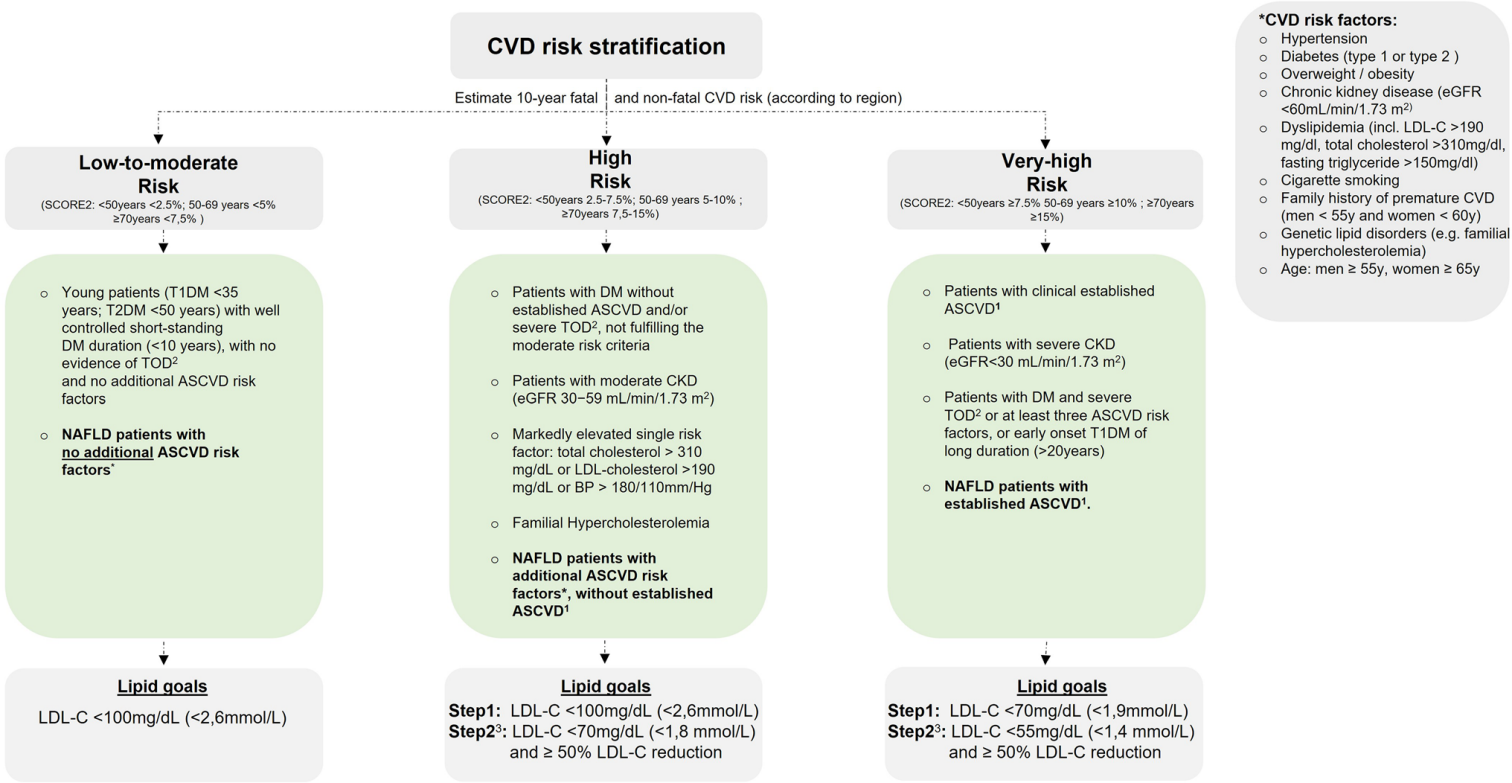
Patient categories with estimated cardiovascular disease risk and assigned lipid goals. (1) Either clinical or unequivocal on imaging. Clinical established ASCVD includes previous ACS (MI or unstable angina), stable angina, coronary revascularization (PCI, CABG, other arterial revascularization), stroke or TIA, aortic aneurysm, and peripheral artery disease. Unequivocally ASCVD on imaging includes significant plaques on coronary angiography, coronary artery CT scan or carotid ultrasound. (2) Target organ damage (TOD) is defined as eGFR < 45 mL/min/1.73 m^2^ irrespective of albuminuria; eGFR 45–59 mL/min/1.73 m^2^ and microalbuminuria (30–300 mg/g); proteinuria (> 300 mg/g); presence of microvascular disease in at least three different sites (e.g., microalbuminuria plus retinopathy plus neuropathy. (3) After Step 1, treatment intensification to the lipid targets of Step 2 should be considered in all patients (taking into account: lifetime ASCVD risk, treatment benefit, risk modifiers, comorbidities, and patient preference). Abbreviations: ASCVD, atherosclerotic cardiovascular disease; BP, blood pressure; CKD, chronic kidney disease; CVD, cardiovascular disease; T1DM, type 1 diabetes mellitus; T2DM, type 2 diabetes mellitus; eGFR, estimated glomerular filtration rate; LDL-C, low-density lipoprotein cholesterol; NAFLD, non-alcoholic fatty liver disease; SCORE2, Systematic Coronary Risk Estimation; TOD, total organ damage

A possible risk stratification algorithm for patients with NAFLD is shown in Fig. 1.

#### Definition of Treatment Targets for Serum Lipids

Based on the risk stratification, individual target values for lipids can be determined.

In patients with NAFLD at moderate risk, an LDL-cholesterol goal of < 100 mg/dL (2.6 mmol/L) should be considered. In patients with NAFLD, who are at high CVD risk, lipid-lowering treatment with an ultimate LDL-cholesterol goal of ≥ 50% LDL-cholesterol reduction and an LDL-cholesterol of < 70 mg/dL (1.8 mmol/L) should be recommended. In patients with NAFLD at very high risk (e.g., with established ASCVD), intensive stepwise lipid-lowering therapy should be sought, ultimately aiming at a ≥ 50% LDL-cholesterol reduction and a target LDL-cholesterol level of < 55 mg/dL (1.4 mmol/L).

#### Lifestyle Modification

##### Body Weight

In patients with NAFLD and elevated BMI and dyslipidemia, guidelines recommend weight loss to achieve and maintain a long-term healthy weight (target BMI 20–25 kg/m^2^, and waist circumference < 94 cm (men) and < 80 cm (women)) as key lifestyle-modifying measure to improve their CVD risk profile [37–39, 48]. In patients with NAFLD, a total body weight loss of ≥ 5% is required to substantial improve hepatic steatosis, ≥ 7% to improve hepatic inflammation or NASH resolution, and ≥ 10% to improve hepatic fibrosis [49–52].

Focusing on dyslipidemia, body weight loss also reduces total cholesterol and LDL-cholesterol levels and a decrease in LDL-cholesterol concentrations of 8 mg/dL (0.2 mmol/L) is observed for every 10 kg of weight loss in obese patients [40]. Serum triglycerides levels are much more responsive to weight changes than serum cholesterol. Even a modest weight loss of 5% has been shown to reduce serum triglycerides levels by around 10% [53]. However, weight loss always needs to be accompanied by further lifestyle modifications such as dietary changes and physical activity interventions to reduce CVD risk significantly.

A beneficial role of physical activity has been demonstrated in numerous studies and is established in all guidelines for both NAFLD and ASCVD [37–39, 41, 52, 54, 55]. Current European guidelines for NAFLD treatment recommend 150–200 min/week of moderate-intensity aerobic physical activities in three to five sessions (e.g., brisk walking, stationery cycling) [38]. Guidelines on ASCVD prevention propose 150–300 min/week of moderate-intensity or 75–150 min/week of vigorous-intensity aerobic exercise to improve cardiometabolic health and reduce CVD morbidity [36, 39]. In addition, resistance exercise training is recommended on 2 or more days per week as well as reduced sedentary behavior, as sedentary time is associated with greater risk for several major metabolic chronic diseases and increased mortality [54, 56].

##### Diet

Several dietary habits such as a high caloric diet, excess of (saturated) fat, sugar-sweetened beverages, high-fructose intake, and refined carbohydrates are associated with weight gain, NAFLD, and dyslipidemia [4, 38, 57]. Especially high-fructose consumption has adverse effects on metabolism leading to the development of intrahepatic insulin resistance, intrahepatic fat accumulation, and the aggravation of an atherogenic lipid profile [58, 59].

Therefore, a healthy diet (e.g., low in saturated fat with a focus on whole grain products, vegetables, fruit, and marine fish) is recommended as a cornerstone of CVD prevention in all NAFLD individuals with dyslipidemia.

The macronutrient composition should be adjusted according to a Mediterranean diet (MD) or a similar dietary pattern [40]. MD is traditionally plant based (whole grains, legumes, fruit, vegetables), low in carbohydrates (limited simple sugars and refined carbohydrates), and rich in monounsaturated (mostly olive oil) and omega-3 fatty acids, and incorporates limited amounts of red meat and low-fat dairy. Although earlier studies have found that calorie restriction rather than the composition has beneficial effects in patients with NAFLD and dyslipidemia, a recent meta-analysis has shown that a MD significantly improves both NAFLD and NAFLD-related CVD risk factors such as hypertension and serum levels of total cholesterol [60].

Daily alcohol intake should be drastically reduced (< 10 g/day for men and women). In patients with hypertriglyceridemia or advanced fibrosis (≥ F2), alcohol consumption should be completely avoided [40].

In conclusion, weight loss in overweight or obese NAFLD with dyslipidemia is urgently needed but insufficient as a single intervention and should therefore always be accompanied by dietary and physical activity interventions.

### Pharmacological Management of Hypercholesterolemia

The causal role of LDL-cholesterol has been demonstrated beyond any doubt in genetic, observational and interventional studies [17, 61–64]. Therefore, lowering LDL-cholesterol is crucial for the treatment of dyslipidemia in patients with NAFLD.

To lower LDL-cholesterol, a stepwise treatment intensification is recommended [39]. Medications that are broadly available and can be used in everyday clinical practice for LDL lowering include statins (inhibiting 3-hydroxy-3-methylglutaryl coenzyme A (HMG-CoA) reductase), ezetimibe (selective cholesterol absorption inhibitor), proprotein convertase subtilisin/kexintype 9 (PCSK9) inhibitors, and bile acid sequestrants. New treatment approaches include bempedoic acid and inclisiran, a new small interfering ribonucleic acid [37, 39].

Statins play a well-established role in the primary and secondary prevention of CVD and are safe among patients with chronic liver disease such as NAFLD, including those with mild baseline elevation in transaminases (< 3 × upper limit of normal [ULN]) or compensated cirrhosis [65–71]. Similarly, there is evidence that statins may attenuate NASH and reduce the risk of liver fibrosis [72].

Although accumulating data show that statins can safely be used in patients with NAFLD, statins are still under-prescribed among these patients due to concerns about hepatotoxicity [73]. However, it should be noted that statins are contraindicated in patients with decompensated cirrhosis or acute liver failure [74, 75].

In addition to their lipid-lowering effects, statins may have beneficial biologic effects, including amelioration of endothelial dysfunction, increased nitric oxide bioavailability, antioxidant properties, and inhibition of inflammation ultimately resulting in improved vascular function and stabilization of atherosclerotic plaques [75, 76].

Treatment with the maximally tolerated dose of high-intensity statins (e.g., rosuvastatin or atorvastatin) is recommended as first-line treatment of dyslipidemia in patients with NAFLD to reach the LDL-cholesterol goals set for the determined risk group [39]. High-intensity statin therapy reduces LDL-cholesterol by 50% on average. For patients with NAFLD and transaminase elevations > 3 × ULN, a lower initial dose of statins and monitoring of transaminases in 4- to 12-week intervals during cautious uptitration may be reasonable [74, 75].

If the therapeutic goal cannot be achieved with the maximum tolerated statin dose, combination treatment with ezetimibe is recommended. Ezetimibe inhibits the intestinal cholesterol absorption through binding to the Niemann-Pick C1-Like 1 (NPC1L1) sterol receptor, which subsequently decreases the transportation of free fatty acids and non-esterified cholesterol to the liver [77]. The average LDL-cholesterol reduction using a combined treatment with a high-intensity statin together with ezetimibe is approximately 65%.

While the use of ezetimibe is safe in patients with NAFLD, there are controversial data on whether ezetimibe can also directly improve biochemical and histological markers of NAFLD [78–83]. For patients not achieving the LDL-cholesterol goals with a maximum tolerated dose of a statin und ezetimibe, combination with a PCSK9 inhibitor is recommended for secondary prevention and should be considered as primary prevention for patients at very high CVD risk. Moreover, a statin combination with a bile acid sequestrant (e.g., cholestyramine) may be considered if the LDL-cholesterol goal cannot be reached. If a statin-based regimen is not tolerated at any dose (e.g., due severe myopathy), a monotherapy with ezetimibe or combination therapy with ezetimibe and PCSK9 inhibitor should be considered [39]. The general principle of managing elevated cholesterol levels follows the rule “the lower the better,” with no adverse effects seen with even the lowest values of LDL-cholesterol [74, 84, 85]. Therefore, there is no need to de-intensify treatment in those who attain very low LDL-cholesterol levels during treatment [74].

**Fig. 2:**
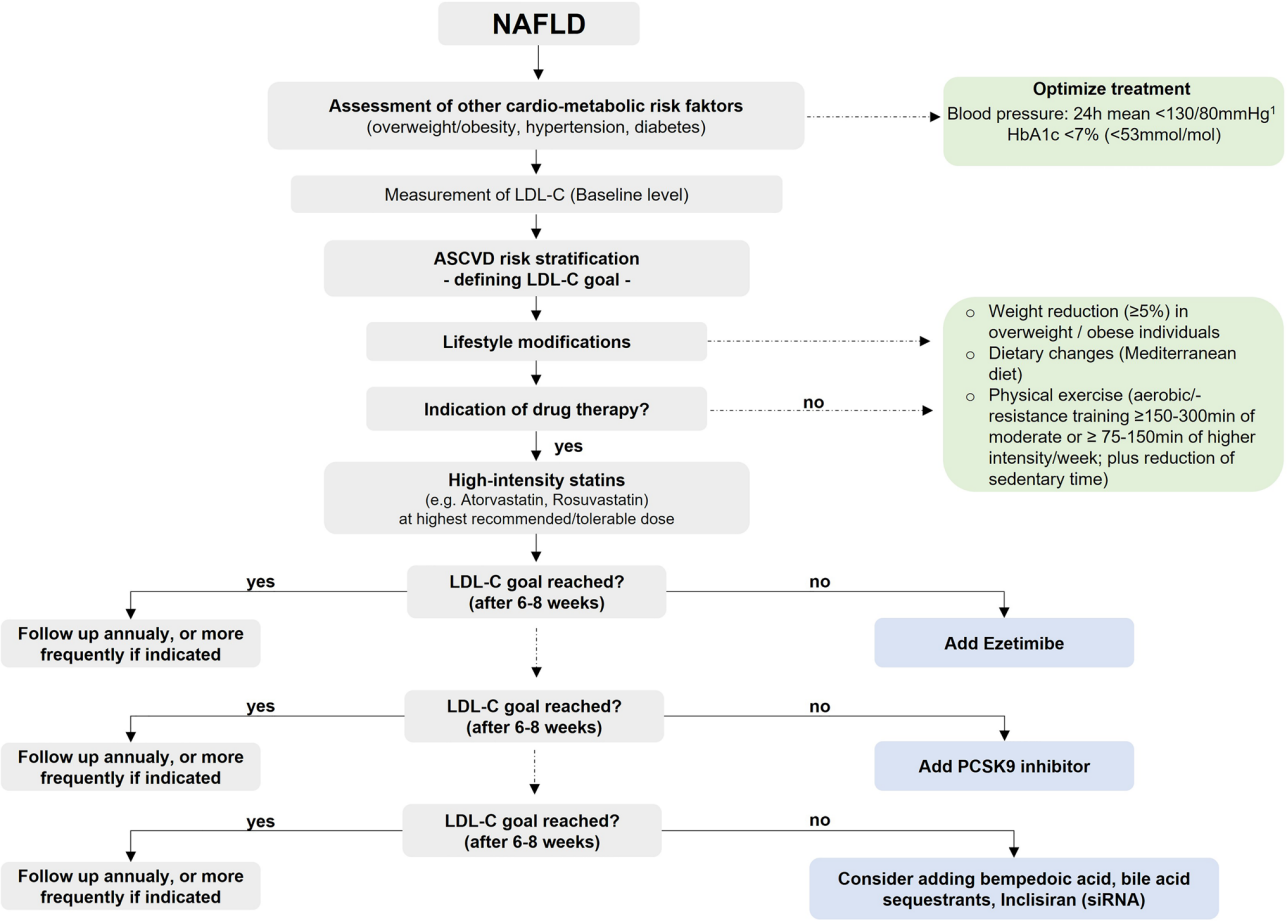
A proposed algorithm for the management of hypercholesterolemia in patients with NAFLD. (1) Blood pressure status should be assessed using 24-h ambulatory blood pressure monitoring. Abbreviations: ASCVD, atherosclerotic cardiovascular disease; HbA1c, glycated hemoglobin; LDL-C, low-density lipoprotein cholesterol; NAFLD, non-alcoholic fatty liver disease; PCSK-9, proprotein convertase subtilisin/kexin type 9; siRNA, small interfering ribonucleic acid

Figure 2 proposes a possible algorithm for the management and treatment of dyslipidemia in patients with NAFLD.

### Pharmacological Management of Hypertrigylceridemia

Although ASCVD risk is already increased at fasting triglycerides higher than 150 mg/dL (1.7 mmol/l), the use of drugs to lower triglyceride levels may only be considered in high-risk patients if triglycerides are higher than 200 mg/dL (2.3 mmol/L) and triglycerides cannot be lowered by lifestyle measures [40, 86]. In addition to the effects on the ASCVD risk, therapeutic lowering of severe hypertriglyceridemia is also useful to reduce the pancreatitis risk, which is clinically significant if triglycerides are > 880 mg/dl (> 10 mmol/L) [40].

In individuals with hypertriglyceridemia [triglycerides > 200 mg/dL (2.3 mmol/L)] and high ASCVD risk, statin treatment is recommended as the first drug of choice.

Statin treatment reduces serum triglycerides by 15–30%, and more importantly VLDL as well as other apolipoprotein B containing atherogenic remnant particles that are typically increased in hypertriglyceridemia patients [53].

In high-risk (or above) patients with triglycerides > 1.5 mmol/L (135 mg/dL) despite statin treatment and lifestyle interventions, the administration of long chain n-3 polyunsaturated fatty acids (PUFAs, e.g., icosapent ethyl, 2 × 2 g/day) may be considered in combination with a statin [39]. Depending on the baseline triglyceride concentration, therapeutic administration of n-3 PUFAs can result in a 30–50% reduction in serum triglycerides [53].

In patients with combined dyslipidemia already taking statins, who achieved their LDL-cholesterol goal, but still display hypertriglyceridemia > 200 mg/dL (2.3 mmol/L), additional treatment with fenofibrate or bezafibrate may be considered. In patients with NASH and dyslipidemia, previous studies investigating the effects of the peroxisome proliferator–activated receptor (PPAR) agonists bezafibrate and fenofibrate have demonstrated beneficial effects on both lipid metabolism and liver function. Fenofibrate treatment in patients with NASH led to a decrease in elevated aminotransferases (ALT, AST) and gamma-glutamyltranspeptidase (GGT) activity as well as hepatocellular ballooning evaluated by biopsy, while short-term treatment with bezafibrate (2–8 weeks) has been found to reduce microvesicular steatosis [87]. However, no significant changes in inflammation and fibrosis could be observed. While the effect of bezafibrate and fenofibrate should therefore be considered as minor, results from studies investigating clinical effects of the new pan-agonist lanifibranor on NAFLD severity and concomitant dyslipidemia will be interesting [88, 89]. Recently published data from a phase 2b study have already indicated promising beneficial effects on improving both NAFLD and dyslipidemia [90].

When focusing on PUFA’s as additional treatment option, it should be recognized that there are controversial results regarding the use of n–3 fatty acid supplementation and their effects on CVD outcomes [91–94]. While the REDUCE-IT trial showed that the use of icosapent ethyl 2 g twice daily was superior to mineral oil in reducing triglycerides, CVD events, and CVD death among patients with high triglycerides [92], the recently published STRENGTH trial, which analyzed the effect of high-dose omega-3 fatty acids (combined formulation of eicosapentaenoic acid and docosahexaenoic acid) in patients at high CVD risk, observed no beneficial effects of omega-3 fatty acid supplementation on the reduction of major adverse CV events [94]. However, it could be observed in both studies that high-dose omega-3 fatty acid supplementation was associated with an increased risk of developing new-onset atrial fibrillation [92, 94]. As NAFLD represents another emerging risk factor for cardiac arrhythmias [95–97], these potential side effects should always be critically considered when PUFA’s are prescribed. In addition, while hepatic de novo lipogenesis is suppressed and fat oxidation is increased by omega-3 fatty acid supplementation, fasting and postprandial glucose concentrations are increased with questionable long-term effects [98].

**Fig. 3:**
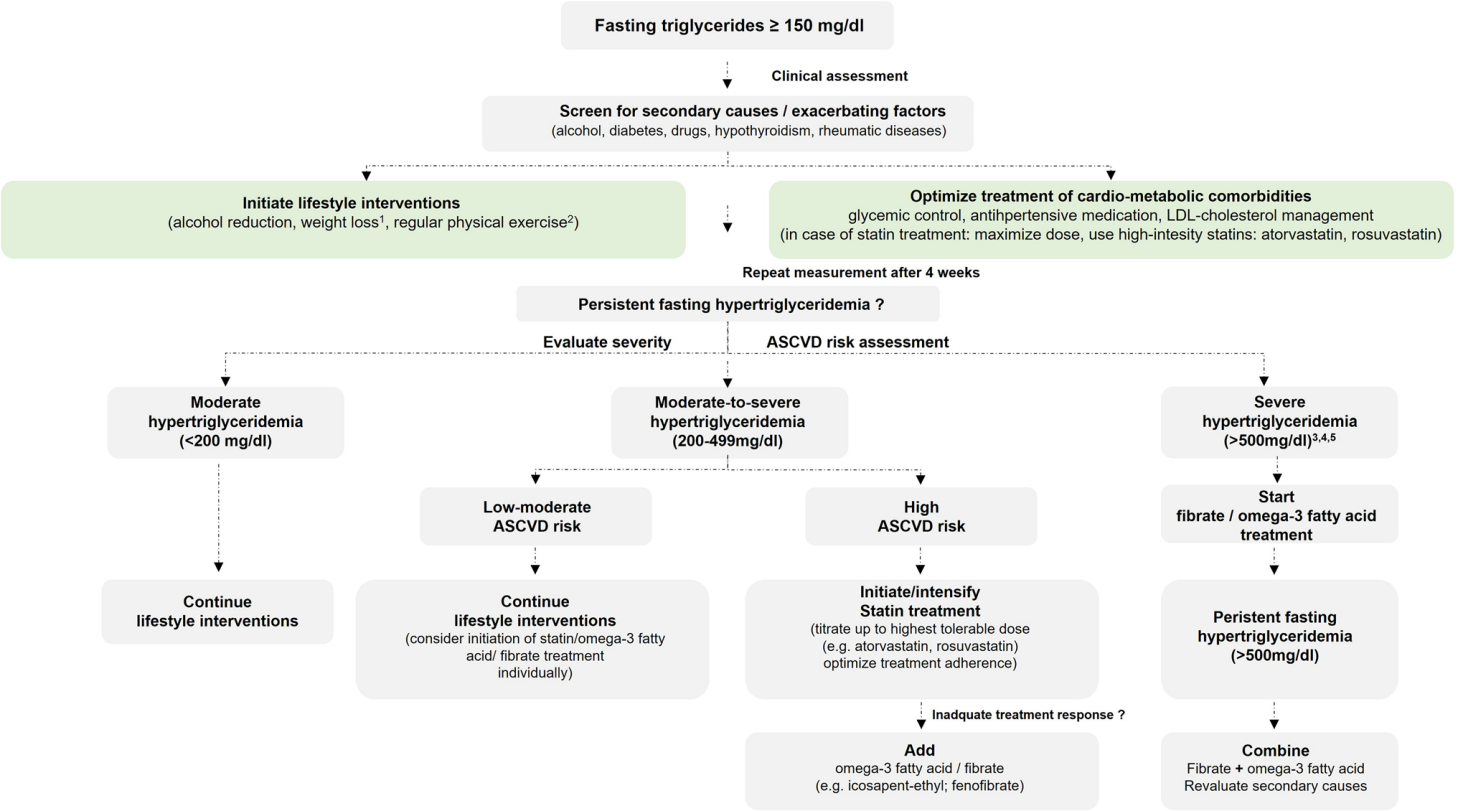
Suggested flowchart for a step-by-step management approach for patients with NAFLD and hypertriglyceridemia. (1) 5–10% for all patients with elevated serum triglycerides; (2) at least 150 min/week aerobic activity at moderate intensity; (3) initiate or increase statin therapy in patients with at least moderate ASCVD risk; (4) emphasize low-fat diet; (5) some expert panels recommend starting fibrate therapy at a triglyceride level of > 880 mg/dl. Abbreviations: ASCVD, atherosclerotic cardiovascular disease

Figure 3 proposes a possible algorithm for the management and treatment of hypertriglyceridemia in patients with NAFLD.

### Treatment of Cardiometabolic Comorbidities

Adequate management of dyslipidemia in patients with NAFLD can only succeed, if all cardiometabolic comorbidities are considered and holistic approaches are needed [99].

Hypertension, obesity, NAFLD, and T2DM commonly coexist with dyslipidemia and act synergistically to increase the individuals’ ASCVD risk. In particular, optimal treatment of DM is important in patients with NAFLD and dyslipidemia, as insulin resistance is the primary mechanism leading to lipid derangements [100, 101].

The prevalence of NAFLD in patients with T2DM is approximately 56% and a recent meta-analysis of 11 studies including 8346 patients observed that patients with NAFLD with concomitant T2DM had a twofold increased risk for the manifestation of ASCVD when compared to patients without NAFLD (OR 2.20; 95% CI 1.67–2.90) [2, 102]. Recently, some anti-diabetic drugs, especially glucagon-like peptide-1 receptor agonists (GLP-1RA) and sodium–glucose cotransporter-2 inhibitors (SGLT-2i), have shown promising results in patients with NAFLD and in ASCVD, with partly beneficial effects on underlying lipid disorders [103–106]. In a recent phase IIb trial in patients with NASH, administration of semaglutide led to higher rates of NASH resolution and no worsening of fibrosis compared with placebo, which was accompanied by favorable effects on triglyceride levels at the highest administered dose [104].

Studies on SGLT-2i in patients with NAFLD showed a decrease in hepatic fat, improved liver function tests, and decreased triglyceride levels, while results on LDL-cholesterol levels were inconsistent [107, 108].

When treating arterial hypertension in patients with NAFLD and dyslipidemia, it should be taken into account that some antihypertensive drugs may have an adverse effect on plasma lipid levels (e.g., thiazide diuretics, beta blockers), whereas others have neutral or beneficial effects (e.g., angiotensin-converting enzyme inhibitors, angiotensin receptor blockers, calcium channel blockers, selective alpha-1 blockers) [109].

### Emerging Drugs for Treating Dyslipidemia in Patients with NAFLD

In recent years, new classes of lipid-lowering agents have been developed and approved that will be of increasing importance in everyday clinical practice in the future.

#### Bempedoic Acid

Bempedoic acid decreases LDL-cholesterol levels by the inhibition of adenosine-triphosphate (ATP) citrate lyase in the liver. ATP-citrate lyase is a cytosolic enzyme upstream of the HMG-CoA reductase in the cholesterol biosynthesis pathway. Unlike statins, bempedoic acid is administered as a prodrug and is converted to its active form by enzymes found only in the liver and not in skeletal muscles. The lack of active metabolites of bempedoic acid in skeletal muscles makes it a promising alternative for patients with statin-associated myopathy. Decreasing LDL-cholesterol synthesis with bempedoic acid leads to an attenuation of atherosclerosis [110]. Bempedoic acid monotherapy and the combination therapy of bempedoic acid with ezetimibe are approved for the treatment of adults with familiar hypercholesterolemia in adults or patients with established ASCVD who require additional LDL-cholesterol lowering after maximally tolerated statin therapy or statin intolerance [111]. Bempedoic acid in a dose of 180 mg daily reduces LDL-cholesterol by up to 20% from baseline either as monotherapy or in combination with statin use. Combination treatment of 180 mg bempedoic acid with 10 mg ezetimibe daily, LDL-cholesterol can be reduced by 50% [74, 112, 113]. However, there is no data from studies investigating bempedoic acid in patients with NAFLD. In addition, trials investigating major cardiovascular endpoints are missing and need to be performed in the future.

#### PCSK9 Inhibitors

PCSK9 protein is an important regulator of circulating LDL‐cholesterol levels. Secreted PCSK9 binds to the LDL receptor on hepatocytes, leading to an internalization and degradation of the receptor in lysosomes. This leads to a reduction of the LDL receptor numbers on the hepatocyte cell surface and a reduced uptake of low-density lipoproteins. In turn, inhibition of PCSK9 in turn increases the number of LDL receptors and an increase uptake of LDL‐cholesterol into cells [114]. The average reduction in LDL-cholesterol with PCSK9 inhibitor therapy is approximately 60% [39, 40]. In combination with high-intensity or maximum tolerated statins, PCSK9 inhibitors reduce LDL-cholesterol up to 75%, and up to 85% when ezetimibe is also added [39, 115, 116]. Further, PCSK9 inhibitors can additionally lower triglycerides [115, 116]. Currently, there are two PCSK9 inhibitors available in clinical practice, alirocumab and evolocumab. Both are administered subcutaneously and lower LDL-cholesterol levels in patients who are at high or very high CVD risk, including those with DM, and induce a substantial reduction in ASCVD events. However, they are currently indicated as rescue therapy for otherwise untreatable patients with severe hypercholesterolemia [117, 118].

Recently presented results from the HUYGENS trial could also demonstrate that treatment with evolocumab had incremental benefits on high-risk features of coronary artery plaques and significantly improved plaque stability by increasing the fibrous cap thickness as measured by optical coherence tomography [119].

Since patients with NAFLD frequently suffer from coronary heart disease with vulnerable coronary plaques [120], the reported positive effects of PCSK9 inhibitors on plaque stabilization seen in the HUYGENS trial may also be of particular relevance in this ASCVD-risk population [121].

While comprehensive studies with long-term follow-up on the effect of PCSK9 inhibitors on the clinical course of patients with NAFLD are lacking, preliminary data suggest beneficial effects and indicate that PCSK9 inhibitors may ameliorate NAFLD via different mechanisms [122]. In a small retrospective study, PCSK9 inhibitors led to an amelioration of hepatic steatosis in patients with NAFLD as measured by computer tomography [123]. However, prospective studies are needed to validate these results.

#### Small Interfering RNA (siRNA) Molecules

Inclisiran, a small interfering RNA (siRNA) molecule, is a novel promising agent for the management of hypercholesterolemia which increases the number of LDL receptors in the hepatocyte membranes by blocking the transcription of PCSK9. It provides advantages because of an infrequent dosing interval of only twice a year to reduce LDL-cholesterol by 50 to 60% [124, 125]. However, currently, no data in patients with NAFLD has been published.

## Conclusion

The concept of NAFLD as a cardiovascular risk factor on its own has been challenged in a study of the Danish general population using a Mendelian randomization design as well as a large matched cohort study including 18 million European adults where an increased risk did not persist after adjusting for established cardiovascular risk factors [126, 127]. As recently pointed out, increasing rates of antihypertensive and lipid-lowering drug treatment in large published cohort studies of patients with NAFLD have been associated with substantially lower cardiovascular mortality [46]. A holistic approach including practical recommendations for lifestyle interventions, diabetes control, blood pressure treatment goals, and risk-related LDL-cholesterol targets seems to be promising. There are no prospective randomized studies looking for effects of lipid-lowering treatments on hard cardiovascular endpoints in NAFLD populations. However, based on the large body of evidence for a significant reduction in cardiovascular morbidity and mortality, it is reasonable to follow the treatment algorithms laid down in current guidelines on lipid modification to reduce the cardiovascular risk in dyslipidemic patients with NAFLD [39, 40].
